# EHMTI-0300. Clinical application of korean version of the international classification of headache disorders, 3rd edition, beta version in university hospitals

**DOI:** 10.1186/1129-2377-15-S1-D6

**Published:** 2014-09-18

**Authors:** S Cho, K Park, H Moon

**Affiliations:** 1 Neuology, Hallym University, HwaSeong-Si, Korea; 2 Neuology, Chung-Ang University College of Medicine, Seoul, Korea; 3 Neuology, Sungkyunkwan University School of Medicine, Seoul, Korea

## Introduction

International Classification of Headache Disorder, an essential tool in the diagnosis of headache disorder, has been revised as 3rd Edition, beta (ICHD-3β).

## Aims

Clinical application in real practice is needed to test a feasibility and usefulness of ICHD-3β with a Korean version.

## Method

The neurologists prospectively enrolled the first-visit patients with headache as a chief complaint from February to March in 2014. Classification of headache disorder was done by each investigator according to ICHD-3β based with the initial structured questionnaire, clinical history, physical examination, laboratory, and neuroimaging studies, if needed. Consensus meeting dealt the cases with any difficulty in diagnosis by each investigator. The feasibility and usefulness were assessed by the proportion of unclassified headache disorders using ICHD-3β and the previous version.

## Result

A total of 207 patients were enrolled: mean age of 41 years (16-87 years), woman 63.3%. A total of 166 patients had primary headache disorders (80.2%): 40.1% migraine (without aura 27.5%, with aura 3.9%, chronic migraine 3.9%, etc), 16.9% tension-type headache (probable 7.2%, frequent episodic 6.8%, etc), 1.4% cluster headache and 21.7% other primary headache disorders. Thirty-five patients (16.9%) had secondary headache disorder and 6 patients (2.9%) could not be classified by ICHD-3β. The diagnoses differ compared to the previous version in 32 patients (15.5%): 14.5% due to mitigation of the previous strict criteria and 1% due to new diagnostic category.

## Conclusion

Classification by ICHD-3β can be done in more than 97% of first-visit headache patients and ICHD-3β is more useful than the previous version.

No conflict of interest.

